# Observations on Polypus of the Uterus

**Published:** 1829-04-01

**Authors:** 


### OBSERVATIONS ON POLYPUS OF THE UTERUS. By *John Macfarlane*, M.D.*

A long, but an interesting, indeed valuable paper on uterine polypi has been published by Dr. Macfarlane, in the fourth Number of our respected cotemporary of Glasgow. The subject is so little understood by practitioners in general that we think we shall be doing some service to the state by noticing the present contribution very fully.

Dr. M. remarks, and we think with reason, that polypi are not so rare as they are thought to be, even by experienced and able practitioners. This, although owing at times, no doubt, to a want of either knowledge or care in discriminating between various morbid conditions of the uterine passages, accompanied as they are by general symptoms of a similar stamp, is partly to be attributed to the delicate nature of the case, the feelings of the patient, and the natural repugnance generally felt to any mechanical or manual examination. This latter, however, is absolutely necessary, and if it were oftener adopted "in women liable to habitual hæmorrhages, and to mucous, sanious, or watery discharges from the vagina, polypus would certainly be found to prove a more frequent disease." Dr. Macfarlane's object being rather to discuss the more important topics suggested by the cases which have come within the sphere of his own observation, than to write an elaborate account of the disease, he has merely considered uterine polypi under two heads—the simple or benign, when the uterus itself is free from disease, and the cancerous or otherwise malignant, complicated or not with organic disease of the womb. These distinctions are made without reference to the part of the uterus from which the polypus takes its rise.

### COMMON OR FIBROUS POLYPUS.

Polypus of the uterus is commonly firmer and more fibrous in its texture than polypus of any other organ, which perhaps may depend on the thickness and vascularity of the uterine substance, the condensed structure of the mucous membrane, or the intimate adhesion of the latter to the parts below opposing much resistance to the morbid growth. The frequent and often profuse hæmorrhages which take place in these cases from the vagina, have by many been attributed to the pressure of the os uteri on the neck of the tumour, impeding the free return of the blood, and causing engorgement and rupture of the superficial vessels. This, together with the pendulous position of the tumour and nature of the parts with which it is connected, will probably fully account for the circumstance. Authors, however, have attempted to explain in a similar manner the pyriform shape and narrow neck of the polype itself, but to this it may be answered that polypi, wherever they are found, assume more or less the above conformation, and cases not unfrequently occur where these tumours are confined to the cavity of the uterus, but possess notwithstanding the pyriform shape and narrow stalk. Dr. Macfarlane has witnessed three such cases, and several preparations illustrative of the fact. At the same

* Glasgow Med. Journal, No. IV.

time it is clear, that when once the tumour has passed the os uteri into the vagina the inferior portion must enjoy in that part more room for expansion than when "cabin'd, cribb'd, confined" in the womb.

Unless the polype has emerged from the uterus, we have no correct means of ascertaining its presence, though of course from the bleeding and accurate examination of the state of the parts, a conjecture may sometimes be formed. In the following cases an accurate opinion was formed, and the ergot of rye was employed with success.

*Case.* The widow of a sea-captain who had had for some years an habitual leucorrœa, was subject to profuse and almost daily discharges of blood from the vagina for the space of ten months, at the end of which time she cousulted our author. Her health being impaired she was ordered to maintain the recumbent position, and the treatment consisted in the administration of mild laxatives, enemata, acids internally, cold to the abdomen, astringent injections into the vagina, and ultimately the introduction of a plug. At the end of three weeks, no benefit having ensued, a manual examination was requested and obtained. The os uteri admitted the point of the finger, but nothing could be felt to indicate a tumour or other disease. The uterus, however, appeared to be enlarged, and its walls bulged out, which induced Dr. M. to suppose that a polypus or some other tumour existed within, and led him to exhibit the ergot of rye, The character of the patient was so highly respectable, and the symptoms and history of the uterine affection such, that our author had no reason to believe she was pregnant, and therefore anticipated no bad effects from the use of the remedy. The first trial failed, as the ergot was apparently bad, but the second occasioned severe and continuous pains, followed by the projection of a smooth, firm tumour at the os uteri, about the size of a small melon. In the course of a few hours the bulk of the tumour had passed into the vagina, and its slender stalk could be felt with the finger. In consequence of the pain and irritation excited, some doses of laudanum were required, but four days after the exhibition of the ergot, the polypus fell off when rising to make water. She had two slight returns of flooding, and in a few weeks was restored to her former good health. The ergot was prepared by infusing a drachm in four ounces of boiling water, and administering one ounce every two hours. Four doses were given before the effect was produced.

"The known efficacy of the ergot in exciting uterine contraction, will readily suggest, in cases analogous to the above, the employment of this medicine, in preference to every other means, whether medicinal or mechanical. It is certainly superior in every respect to a practice alluded to by Gardien, in his "Traité d'Accouchemens," tome i. p. 430, in which a M. Bonnie dilated the os uteri by means of sponge tents, and afterwards applied a ligature to the polypus.

"A spontaneous cure is sometimes effected when the pedicle is small, either by the neck of the uterus firmly compressing it; or more frequently the propulsive efforts of the uterus, by stretching the pedicle, causes it to ulcerate. This termination is by no means so common, however, as many authors would have us to believe. I have had frequent opportunities of examining tumours thus expelled, several of which were pronounced to be polypi; but after careful examination, the one above referred to was alone entitled to be considered as a specimen of this disease. The others were either the fungous excrescences, denominated "vivaces" by Levret, and supposed to arise from an ulcerated spot in the cavity of the uterus; or more generally fleshy moles, now believed to be the product of conception. The true polypus which was separated by the action of the uterus, was of a firm texture, covered by a mucous membrane, and weighed about 6ozs. Its substance was dense and homogeneous, showing only a slight fibrous appearance about the pedicle; a small portion of which was attached, and about the size of a quill. The one half of this specimen was carefully dissected, and the other macerated, but without throwing any light on its structure or mode of growth. The mucous coat, after a few days, was easily separated, and the great bulk of the tumour that remained, broke down under the fingers, showing in some parts a lamellated arrangement of its texture. In this, as in every other specimen of uterine polypus which I have had an opportunity of examining, no very obvious disease of the mucous membrane was discoverable. It was sometimes found slightly thickened, irregular, and traversed by enlarged veins, but was always separable from the surface of the tumour; and it never appeared as if the whole bulk of the polypus had depended 'on a morbid change of that membrane, and a slow subsequent enlargement of the diseased portion;' a circumstance which Dr. Burns, in his excellent 'Principles of Midwifery,' considers as the most frequent variety of the disease. From whatever part or texture of the uterus the disease originates, it is probable that in a great majority of cases the mucous tissue of this viscus is not its primary seat; but that, in consequence of the tumour being developed behind it, it is gradually pushed forward, becomes elongated, and is afterwards liable to slight morbid changes, arising chiefly from mechanical distention or chronic inflammation. There is reason to believe, however, that in very large and old polypi, particularly in those which have a tendency to assume a malignant action, the mucous envelope will be found so much changed and assimilated to the morbid mass which it covers, as to have lost its natural appearance and distinctive characters." 416.

From several well-authenticated cases upon record it is proved, that pregnancy is not incompatible with the presence of a polypus of considerable size in the uterus. The following is, in Dr. Macfarlane's opinion, an almost *unique* instance, in many respects, of such a combination.

*Case.* Mrs. S. ætatis 30, was taken in labour of her first child, on the morning of the 13th October, 1825. About 4 o'clock on the succeeding morning the child was born; but its feeble condition and premature appearance tended to corroborate her own opinion that she was hardly eight months gone in pregnancy. In half an hour, the placenta was partly protruded by a slight bearing-down pain; but on Dr. Macfarlane's attempting to remove it, unexpected resistance was encountered, which was found, by examination, to depend on its adhesion to a large, firm, globular tumour that filled the vagina and rested on the perineum. The centre of the placenta, opposite the cord, adhered to the apex, and the rest embraced the sides of the tumour, all which attachments were separated during the examination, and the placenta itself brought away. At first it was thought that the tumour consisted in an inversion of the uterus, but on pushing it up before the hand, an immense firm polypus was found growing from the very centre of the fundus of the uterus, which had caused by its weight and descent with the placenta a partial inversion of that organ. The tumour was of almost cartilaginous hardness, its pedicle as thick as the wrist, and its depending part larger than a child's head at birth; its surface was smooth to the feeling except at the apex, where a roughness was occasioned hy the adhesion of the placenta. From the thickness of its attachment it seemed evident to our author that any attempt to twist it off would more probably occasion laceration of the substance of the uterus than of the pedicle, and therefore any such idea was given up. During the minute and a half that this examination was proceeding, blood was profusely issuing from the apex of the tumour, and, in consequence of the presence of the hand in the vagina, rapidly accumulating in the uterine cavity. The clots were scooped out, and the uterus excited to contract as much as possible on the surface of the polypus, but the hæmorrhage proceeded, and after a few minutes the pulse became indistinct, and the patient complained of approaching syncope. The head was laid low, cold cloths applied to the vulva and abdomen, the windows of the room thrown open, and, in half an hour, the hæmorrhage had ceased. The symptoms, however, were now most alarming, the lips being bloodless, the body cold and clammy, the pulse indistinct, the breathing laborious. Professor Towers was now sent for, and in the interim the cold applications were continued, pressure applied over the fundus uteri, and the patient persuaded to swallow a quantity of undiluted whiskey every three or four minutes, together with which ten drops of laudanum in a glass of hot whiskey and water were administered every ten minutes. At half-past seven Mr. Towers arrived, when the patient was alarmingly exhausted. The stimuli were continued, either whiskey or brandy, joined with laudanum or the black drop, being given as often as the pulse became imperceptible; bottles of hot water applied to the feet, and hartshorn to the forehead and nose. These means were assiduously employed throughout the day with the effect of only rousing her at intervals, till 4, p. m. when the pulse grew more distinct, the breathing more calm, and the countenance less anxious. A drachm of laudanum in a glass of brandy now procured an hour's sleep, after which she awoke with a fuller pulse, some colour on her face, and a warmer skin. Dr. Hugh Wood remained with her during the night, giving stimuli till re-action was fairly established, and next day she was easy, but somewhat excited. She was ordered to be kept cool and quiet, to take only gruel, and to swallow a dose of castor-oil. The antiphlogistic regimen was continued till the 21st, when beef-tea, chicken-broth, arrow-root, &c. were allowed. From the 15th to the 25th, the catheter was obliged to be introduced twice daily, and injections were necessary to correct the acrimony and fetor of the discharge. In three weeks the patient could leave her bed, but for some time she could only void her urine by lying on her back, with the head low, and the breech raised. In three months the tumour had decreased to the size of a small orange, and Dr. Macfarlane has reason to believe that it has not since augmented. She has never subsequently enjoyed good health, is pale and sallow, and looks as if ten or fifteen years had been added to her age. She has at intervals attacks of hæmorrhage, with an almost constant discharge of a thin and dark-coloured fluid, but nothing will induce her to submit to the application of a ligature to the tumour. The following observations on the case are ingenious and just.

"It is probable, as there existed in this case no symptom indicating the presence of uterine disease previous to impregnation, that the polypus, if then developed, must have been extremely small, and that it gradually increased in size in proportion to the growth of the placenta which was attached to it. From the general enlargement and activity of the vessels of the uterus, during the whole period of utero-gestation, it is evident, that every adventitious tumour attached to this organ will, during that period, increase in the same ratio with the uterus itself, from its receiving the same increased supply of blood. This result will, however, be more obvious when, as in this case, the blood destined to the nourishment of the fœtus has to pass and repass through the tumour; and it is to this circumstance we are to attribute in some measure its rapid growth. The great diminution that took place in the polypus soon after delivery, evidently showed that its previous large size depended more on its adventitious attachments to the placenta, than on a gradual augmentation of its substance. I consider the adhesion of the placenta to the tumour alone, and its having no connexion with the uterine surface, as the most curious part of this singular case.

"The quantity of blood lost was small, in proportion to the long continuance and alarming appearance of the symptoms, and the previously robust condition of the patient. I have often seen more blood lost after an ordinary labour without any disagreeable symptom supervening, yet to this source alone are they to be referred; for it was apparent, that had stimulants not been immediately and unceasingly administered, the vital powers would have sunk irrecoverably. It was found, that during the twelve hours' continuance of this treatment, viz. from 5, A.M. to 5, P.M. she had taken about 17oz. of ardent spirits, ℥xj. of laudanum, and nearly half a bottle of the black drop, without deranging the stomach, or inducing the slightest tendency to intoxication. From the firm texture of the tumour, and the great thickness of its pedicle, I am convinced, that although a ligature had been accurately and tightly applied to its neck, the bleeding would not have been arrested; for when I grasped the pedicle as firmly as possible with the hand, it had no effect in restraining the hæmorrhage." 421.

A question arises in the treatment of polypus first discovered immediately after delivery, namely, whether we should attempt its removal by ligature then, or delay for some months till the womb has recovered its natural size. The *pros* and *cons* are so ably and yet so concisely stated by Dr. Macfarlane, that abridgment would be injury.

"It may be argued, in favour of an immediate operation, that from the dilated state of the os uteri and neighbouring parts, the hand can be introduced with ease, the exact attachment of the tumour ascertained, and the ligature applied as near the root as may be necessary, having it thus in our power to avoid including any part of the uterus—that many women who are easily alarmed at the prospect of the most simple operation, would more readily submit during that period of comparative ease and comfort, which immediately succeeds the painful process of parturition, than when the mind has had time to call up the ideal difficulties and dangers which not unfrequently present themselves—and that it might even be applied without the patient's knowledge, and thus the exhausting discharges, and consequent bad health which usually succeed, would be avoided. There are a very few cases on record in which this treatment was adopted with success. Mr. Bell, of Forres, details a case in the Edinburgh Medical and Surgical Journal, vol. xvi. in which a polypus of about six pounds weight was tied, after an instrumental labour, without any disagreeable consequences; and M. Deguise,* after delivering a woman of twins, applied a ligature to the pedicle of a polypus, 'du volume d'une poire de bon chretien,' which separated on the 8th day.

"On the other hand, by delaying the ligature for some months, we expect that an obvious diminution will take place in the size of the tumour, in consequence of a decrease in the size and activity of the uterine vessels, and that we are less likely to encounter urgent or alarming symptoms from this measure, when the uterus has returned to its former volume, than when enlarged, irritable, and its vessels still active—a state of parts known to exist for some time after labour has been completed. Under the most favourable circumstances, this operation is not unfrequently attended by painful and somewhat urgent symptoms; and several cases are recorded by Denman, Levret, and others, in which it proved fatal in some cases from hæmorrhage, and in others by the supervention of peritoneal inflammation. Now, such occurrences are obviously more likely to happen after delivery, than when the disease is unconnected with pregnancy. No doubt, if the pedicle is small, and the woman neither of an irritable habit, nor prone to inflammatory attacks, it may then be safely tied; but as a general rule, we ought to defer this operation until both

"* Nouveau Journal de Médecine, tom. ii."

uterus and polypus have considerably decreased, when it may be undertaken with less risk to the patient. It might be supposed, that as a polypus is not a natural part of the human body, but a morbid growth, no danger could attend a ligature of its pedicle, provided no part of the substance of the uterus was included. But it is impossible to tie an uterine polypus, without including its external covering, which is formed by an elongation of the delicate and sensible membrane that lines the cavity of the womb, and it is to this circumstance that many of the urgent symptoms resulting from this operation are to be referred." 422.

Dr. M. believes that the symptoms becomes less urgent so soon as the mucous coat is divided, the remaining less sensible substance admitting of being compressed with greater impunity. The following case exemplifies the above observation, and likewise proves that the occurrence of ulceration in the tumour by no means ensures its removal or destruction.

*Case.* Dr. M. was summoned, on the 15th March, 1826, to visit a lady just delivered of her third child, in consequence of a large, firm, insensible, and rounded tumour, filling the vagina, and protruding slightly at the external orifice. The placenta was retained, the hæmorrhage moderate, the patient exhausted. In order to excite contraction of the uterus, and expulsion of the placenta, the hand was introduced, and the state of the polypus, for polypus it was, ascertained. It was nearly as large as a child's head at birth, smooth on its surface, and attached to the posterior wall of the uterus, two inches from its orifice, by a pedicle about an inch and a half in circumference, and three inches in length. The pedicle was so soft as to have been torn with ease, but the arteries passing to the tumour were so large that no such procedure was attempted. The placenta was soon detached and expelled, the polypus descended into the vagina, the uterus contracted, and the hæmorrhage gradually ceased. The catheter was required for several days, but three weeks after delivery the polypus had shrunk so much that the finger could be passed around it with ease, and it only gave her pain on assuming the erect position. It was now discovered that several cavities were formed by ulceration in the substance of the tumour, one of them so deep, as to admit the finger to the depth of a quarter of an inch. Her health now suffered in spite of the exhibition of tonics, bitters, chalybeates, &c. and, after the lapse of two months, although the ulceration had stopped, cicatrization of the sores appeared to have occurred, and the bulk of the tumour remained. The ligature was therefore applied on the 26th of June, by means of the double canula, guided along the finger, and introduced within the os uteri. It having been ascertained, by passing the finger around the pedicle, that no portion of the uterus was included, the ligature was gradually tightened, and the ends secured to the canula which hung from the vagina. She complained immediately of pain, and this, in a few minutes, became so severe, that full doses of laudanum were given to allay it. On the 29th, the ligature was tightened again, and excited a slight return of pain, the process was repeated on the 1st of July, and on the 3d, the ligature came away with the canula, and the polypus was extracted with some difficulty, by means of a blunt hook. It weighed 18½ ozs. was of fibrous structure, and its mucous membrane was thickened, purple, and in some places separated and disorganized. In six months afterwards the lady became pregnant, and in due time was delivered of a living child, without any return of the disease.

The practice of twisting off polypi by the forceps, Dr. M. very justly disapproves of, as likely, when the pedicle is large, to give rise to laceration of the womb, a termination which proved fatal, a few years ago, in the practice of a surgeon in the neighbourhood of Glasgow. If, indeed, we were sure that the pedicle was small, the plan might be adopted with success, but this we are often unable to determine, and the practice is, therefore, one which has deservedly fallen into disuse. M. Dupuytren's plan of drawing out the polypus from the vagina, exposing the pedicle, and cutting it across, our author considers "unworthy of imitation." In very relaxed habits, when the disease is combined with prolapsus uteri, and arises from the cervix, he believes that no great force will be required in completing the first stage of this process. "When, however, the uterus retains its natural position, and the root of the tumour is broad, and attached near the fundus, we will seldom succeed in effecting our purpose, unless by tearing away the tumour, injuring the substance of the uterus, or causing its inversion. This latter occurrence is not so likely to happen when the tumour is fixed to the cervix, as when attached near the fundus, which is the most frequent seat of the disease. Besides when the pedicle has been exposed and divided, we will generally find that the uterus at once resumes its former situation in the pelvis, so soon as the extracting force is removed, and that we may afterwards have a hæmorrhage, which we can with difficulty suppress." The fact is, that much also will depend upon the size of the polypus itself. We remember seeing a very eminent surgeon try to bring down by forceps, double hooks, and various instruments, a good-sized polypus through the os externum, without success, indeed with some risk of rupturing the perinæum. When this plan had failed, a ligature was applied round the pedicle in situ, by means of the double canula, with perfect ease. In another instance, where the polypus was small, it was drawn from the vagina, or at least to its orifice, with great facility, and removed by a pair of curved scissors. The polypus, in both instances, was apparently attached to the cervix of the womb.

When difficulty exists in extracting a large polypus, after its pedicle is divided, Dr. Macfarlane believes, that properly directed manual or instrumental aid will succeed in the majority of cases, and should always be employed in preference to allowing a large mass of dead and putrefying matter to remain in an excoriated and irritable vagina. If the entire polypus does not occupy the vagina, but a portion still remains within the uterus, in a situation to be acted upon by its fibres, we may expect that, in some cases, the unaided efforts of Nature will accomplish its expulsion, and at any rate, after a short delay, our author thinks that little danger would result from attempting to excite the action of the uterus by one or two doses of the ergot of rye. Inability to make water, so frequently observed in the progress of the disease, is commonly from sympathy only, occasionally, however, as in the following interesting case, from mechanical obstruction to the urinary flow.

*Case.* A thin and exceedingly deformed woman had a severe attack of pain and swelling of the belly, which disappeared in a single night, after a copious discharge of purulent matter from the vagina, which continued for a long period. Many years after this, and nine months after her marriage, she applied to Mr. Stirling, with great pain and tension in the lower part of the abdomen, and retention of urine. A catheter was with difficulty introduced, on account of an immense tumour in the vagina, which proved to be a polypus, though neither its pedicle, nor yet the os uteri, could be felt. In consequence of frequent attacks of retention, and the size and descent of the tumour, a ligature was applied. She was seized, immediately afterwards, with peritonitis and retention of urine, and it was found, after repeated attempts, that the catheter could not be introduced. As she had an urgent desire to void urine, and as the bladder appeared to be distended, it was agreed in consultation to puncture this viscus above the pubes. A small quantity of urine escaped, and gave her slight relief, but in two days afterwards she died of peritoneal inflammation.

On dissection, the uterus projected into the cavity of the abdomen, and equalled the size of this viscus at the seventh month of utero-gestation. When it was cut out and inverted, the polypus was found to adhere by *three pedicles*, one about the thickness of the thumb, attached to the fundus, and the other two a little distance from the former, and of smaller diameter. The tumour, which was almost cartilaginous in structure, weighed with the uterus upwards of seven pounds. The case is interesting, as shewing that the application of a ligature to these tumours is not to be regarded as entirely free from danger.

"It is extremely rare to find a uterine polypus attached by more than one pedicle, but according to Levret,* is by no means uncommon to find that it contracts adhesions to the cavity in which it is contained. As in the above case there was no appearance at the junction of the body and pedicles, as if the tumour had been originally formed of three separate parts, but as it exhibited a smooth regular appearance, I think that the one attached to the fundus is the proper pedicle, and that the other two have probably arisen from some old adhesions between the tumour and uterus, which had become elongated by the weight and descent of the polypus. It would appear from a report in the Lancet, (No. 244,) that a case lately occurred to Mr. Lawrence, in St. Bartholomew's Hospital, in which it was found, on dissection, that the large pedicle of the polypus was attached "to the whole circumference of the os uteri." This could not have existed six months before her admission, when, in consequence of the tumour presenting in the vagina, she was delivered by the forceps, but I think adhesion had subsequently taken place between the os uteri and pedicle of the polypus, so as to cause this very broad and unusual attachment." 428.

The last division of the paper on malignant polypi we shall touch on but slightly, as, unfortunately, the practical deductions are but few, and art unavailing in remedying or removing the horrible disease. The state of malignancy cannot always be readily discovered; for, as Dr. Macfarlane justly remarks—

"If the uterus is affected about its orifice, the disease tolerably developed, and the constitutional symptoms well marked, we will have little difficulty in our diagnosis; but the consistence of the polypus is seldom calculated to yield us any satisfactory evidence. Many such tumours have been successfully extirpated, without any tendency to reproduction, which, from their hardness, were believed to be scirrhous. Indeed, the structure, on dissection, of a firm fibrous polypus so nearly resembles scirrhus, as to render a mistake by no means improbable. In both forms, the affected portion consists of a hard condensed texture, of a whitish, or slightly yellow colour, irregularly intersected by thick membranous septa. By some authors, the sallow and cachectic appearance of the countenance, the copious discharge of bloody and fœtid matter from the vagina, and the ulceration of the polypus, have been considered as accurate indications of a cancerous degeneration. But although these symptoms may attend this form of disease, they are also frequently present when the tumour is of a benign character, and the uterus unaffected. The unhealthy appearance of the countenance depends on the long-continued irritation, and exhausting discharges; the fœtor of these discharges, on the retention and decomposition of part of the effused blood; while the ulceration of the tumour cannot be considered as an indication of a cancerous action, for superficially ulcerated patches

"* Observations sur plusieurs Polypes de la Matrice. Trois. edit. p. 557."

are not unfrequently found in the mucous texture of these tumours; and occasionally, as in case 4th, the ulceration extends more deeply. If the above symptoms exist in combination with severe lancinating pains in the region of the uterus, there will be grounds for believing that a morbid action has begun either in the uterus or polypus, for the removal of which our best directed efforts will prove unavailing. Still, if no very palpable evidence of malignancy can be obtained by examination, we ought not, even under the above doubtful circumstances, to refuse our patients the chance of an operation." 429.

Two cases are given, in one of which the uterus was extensively diseased, and, in the other, considerably thickened at the part whence the polypus arose; but we need not do more than allude to them here. Our author observes, that when the root of a polypus does not drop off soon after the pedicle has been divided by ligature, a secondary tumour is sometimes formed, possessing an irregular oblong shape, growing more rapidly than the previous polypus, and seldom displaying so dense a structure. This secondary tumour may have its surface smooth and polished by the action of the uterus, but it wants that covering of mucous membrane which always invests a true polypus. The paper ends with the relation of a case, where simple polypus grew from an uterus excessively diseased, and with it ends this full, but we hope not unprofitable, analysis. The subject of uterine polypi has been clearly discussed by Dr. Macfarlane, and the reader of his paper can scarcely fail to derive instruction from its careful perusal.

